# Selenite promotes all-*trans* retinoic acid-induced maturation of acute promyelocytic leukemia cells

**DOI:** 10.18632/oncotarget.12531

**Published:** 2016-10-08

**Authors:** Sougat Misra, Arun Kumar Selvam, Marita Wallenberg, Aditya Ambati, András Matolcsy, Isabelle Magalhaes, Gilbert Lauter, Mikael Björnstedt

**Affiliations:** ^1^ Department of Laboratory Medicine, Division of Pathology F46, Karolinska Institutet, Karolinska University Hospital Huddinge, Stockholm, Sweden; ^2^ Therapeutic Immunology Unit, Department of Laboratory Medicine, Karolinska Institutet, Stockholm, Sweden; ^3^ Centre for Allogeneic Stem Cell Transplantation (CAST), Karolinska University Hospital, Stockholm, Sweden; ^4^ 1st Department of Pathology and Experimental Cancer Research, Semmelweis University, Faculty of Medicine, Budapest, Üllői út, Hungary; ^5^ Department of Oncology-Pathology, Karolinska Institutet, Stockholm, Sweden; ^6^ Department of Biosciences and Nutrition, NOVUM, Karolinska Institutet, Huddinge, Sweden

**Keywords:** acute myeloid leukemia, selenite, all-trans retinoic acid, differentiation, PML/RARA

## Abstract

Selective targeting of the PML/RARα oncoprotein demonstrates a successful molecular targeted therapy in acute promyelocytic leukemia (APL) with a typical t(15:17) chromosomal translocation. The zinc-thiolate coordination is critical for structural stability of zinc finger proteins, including the PML moiety of PML/RARα. Based on the known interaction of redox-active selenium compounds with thiolate ligands of zinc, we herein have investigated the abrogatory effects of selenite alone or in combination with all-*trans* retinoic acid on PML/RARα and the possible effects on differentiation in these cells. At pharmacological concentrations, selenite inhibited the proliferation and survival of APL originated NB4 cells. In combination with ATRA, it potentiated the differentiation of NB4 cells without any differentiating effects of its own as a single agent. Concordant with our hypothesis, PML/RARα oncoprotein expression was completely abrogated by selenite. Increased expression of RAR, PU.1 and FOXO3A transcription factors in the combined treatment suggested the plausible basis for increased differentiation in these cells. We show that selenite at clinically achievable dose targets PML/RARα oncoprotein for degradation and potentiates differentiation of promyelocytic leukemic cells in combination with ATRA. The present investigation reveals the hitherto unknown potential of selenite in targeted abrogation of PML/RARα in APL cells with prospective therapeutic value.

## INTRODUCTION

Acute promyelocytic leukemia (APL) is characterized by clonal expansion and accumulation of hematopoietic stem cells that fail to differentiate into mature leukocytes due to blockade of differentiation at early developmental stages. APL is associated with the t(15;17) (q22;q21) chromosomal translocation in majority of patients [1]. This translocation predominantly leads to fusion of the genes for promyelocytic leukemia (PML) and retinoic acid receptor alpha (RARα), resulting in the translation of fusion proteins PML/RARα and RARα/PML [2, 3]. The fusion proteins harbor the DNA and ligand binding domains (LBD) of RARα [4]. It is envisaged that these fusion proteins abrogate DNA binding of native RARα in a competitive manner. This is followed by transcriptional inhibition of RARα-mediated gene expressions that are important for the lineage-specific terminal differentiation of immature blast cells [5]. These fusion proteins also share common DNA binding sites with PU.1, an important transcription factor for myeloid and lymphoid cells differentiation [6, 7], and thereby compete and interfere with the hematopoietic cell differentiation [4].

Treatment with all-*trans* retinoic acid (ATRA) and arsenic trioxide (ATO) have dramatically improved the survival of APL patients with higher percentage of complete remission [3]. ATRA exerts its effects by binding to the LBD of PML/RARα, eventually leading to the degradation of the C-terminal domain of the chimeric protein in a caspase-dependent manner [8]. In contrast, ATO targets conserved cysteine residues in the zinc finger domain of the PML subunit of PML/RARα, resulting in PML oligomerization and subsequent degradation of the complex by SUMOylation [9]. In combination, both compounds diminish the repressive effects of PML/RARα, while potentiating the RARα and PU.1-mediated maturation. Nevertheless, ATRA/ATO-induced clinical remissions are often associated with differentiation syndrome [10] along with systemic inflammatory response syndrome, increased activity of cytochrome P-450, upregulation of multidrug resistance protein 1 (MDR1), inhibition of thioredoxin reductase and a blunted effect of ATRA following the mutation of PML/RARα in the LBD of certain leukemic clones [3].

As indicated above, targeted degradation of PML/RARα represents an established molecular-targeted mechanism for curing APL. Herein, we have conceived a similar mechanism of action by a redox-active selenium compound, selenite, implicated in the removal of zinc from zinc/thiolate coordination sites [11]. Experimental evidence on selenite-mediated inhibition of DNA binding activity of zinc finger transcription factor SP1 and release of zinc [12] are congruent with the proposed mechanism. Furthermore, signaling pathway analyses reveal the fundamental basis for the potential use of selenite in the treatment of APL. Selenite induces the expression of transcription factor FOXO3A which plays an important role in ATRA-induced differentiation in NB4 cells [13]. Furthermore, in prostate cancer cell (LNCaP) and in Friend erythroleukemia cells, selenite inhibits the activity of DNA methyltransferase (DNMT) [14, 15], a known inducer of leukemogenic potential in APL upon recruitment by PML/RARα [16]. Apart from targeting the above-mentioned molecular pathways implicated in impeding differentiation in APL cells, redox-active selenium compounds, including selenite, comprise a novel class of cancer chemotherapeutic agents with superior cytotoxic effects on many cancer cells including those of leukemic origin. In an earlier study, we have reported that primary acute myeloid leukemia (AML) cells are more sensitive to selenite at pharmacologically achievable doses [17] compared to conventional anti-leukemic drugs at clinically relevant concentrations [18]. It has also been shown that selenite is a potent inhibitor of growth and survival of APL-originated NB4 cells *in vitro* [19], with autophagy/apoptosis being the major cell death pathway [20]. These observations together led us to examine the potential roles of selenite alone or in combination with ATRA on growth inhibition and differentiation in NB4 cells. Herein, we provide evidence that ATRA in combination with selenite at pharmacologically achievable doses diminish the survival and proliferation of these cells, with enhanced maturation in the surviving cell population in comparison to ATRA alone.

## RESULTS

### Cell proliferation and viability upon treatment with selenite and ATRA

**Figure 1:**
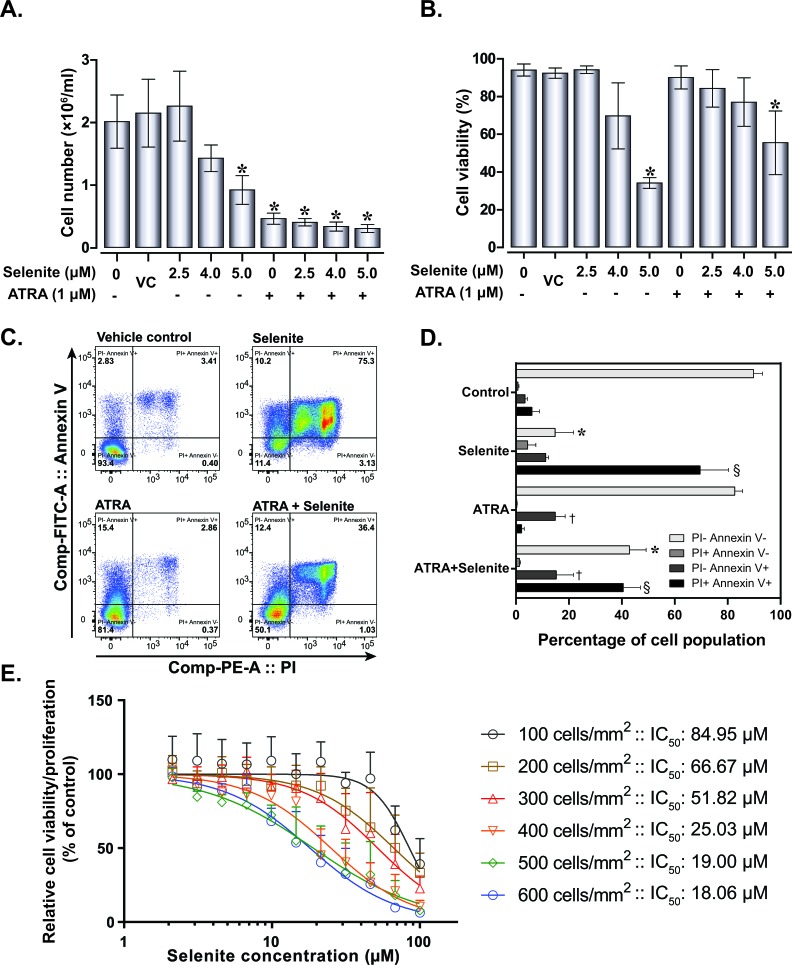
Effect of selenite and ATRA on cell viability and proliferation Cell proliferation **A.** and viability **B.** of NB4 cells following single or combined exposure to ATRA and selenite for 120 h (*n* = 5-10). VC - vehicle control; (*) indicates significant differences among the treatment groups compared to vehicle control. **C.** Representative flow cytometry analysis for apoptosis in NB4 cells following 120 h treatment and **D.** quantitative analyses (*n* = 3). Mean values with different symbols indicates significantly different from the vehicle control group. **E.** Cytotoxicity of selenite as a function of cell density. NB cells were seeded at different density and selenite cytotoxicity was evaluated after 24 h by WST-1 assay (*n* = 3). Error bars represent ±/+ standard deviation.

Initially, we examined cell proliferation and viability to investigate the dose-response effects of selenite alone or in combination with ATRA. NB4 cell proliferation was diminished with increasing selenite concentrations (Figure 1A). Consistent with previous studies, ATRA exerted significant anti-proliferative effects in these cells. A significant reduction of cell viability (mean viability 34.27%, confidence interval of mean 2.83%) was observed following treatment with 5.0 μM selenite (Figure 1B), while treatment with 1.0 μM ATRA alone induced no appreciable toxicity. Nevertheless, we observed reduced cytotoxicity (mean viability 62.44%, confidence interval of mean 13.36%) in the combined treatment at the highest selenite concentration. To characterize the nature of cell death processes involved, we further performed flow cytometry analyses of propidium iodide - Annexin V stained cells under identical experimental condition (Figure 1C and Figure 1D). We found substantial apoptosis in NB4 cells following treatment with selenite. In the presence of ATRA, the cytotoxic effect of an equivalent dose of selenite was diminished. However, a significant cell population underwent apoptosis. These findings were congruent with the trypan blue exclusion assay. To further investigate the diminished cytotoxicity of selenite in the presence of ATRA, we measured the effect of selenite on cell viability in a time course of 24h (Figure 1E). This was also performed to demonstrate how differences in seeding densities and cell proliferation influence selenite toxicity, as we consistently observe that selenite cytotoxicity greatly depends on cell density. We found that the relative 24 h IC_50_ of selenite ranged between 18.06 - 84.95 μM depending on the seeding density of cells, with the highest cytotoxicity observed at the highest seeding density (Figure 1E).

### Selenite in combination with ATRA promotes NB4 cells differentiation

**Figure 2:**
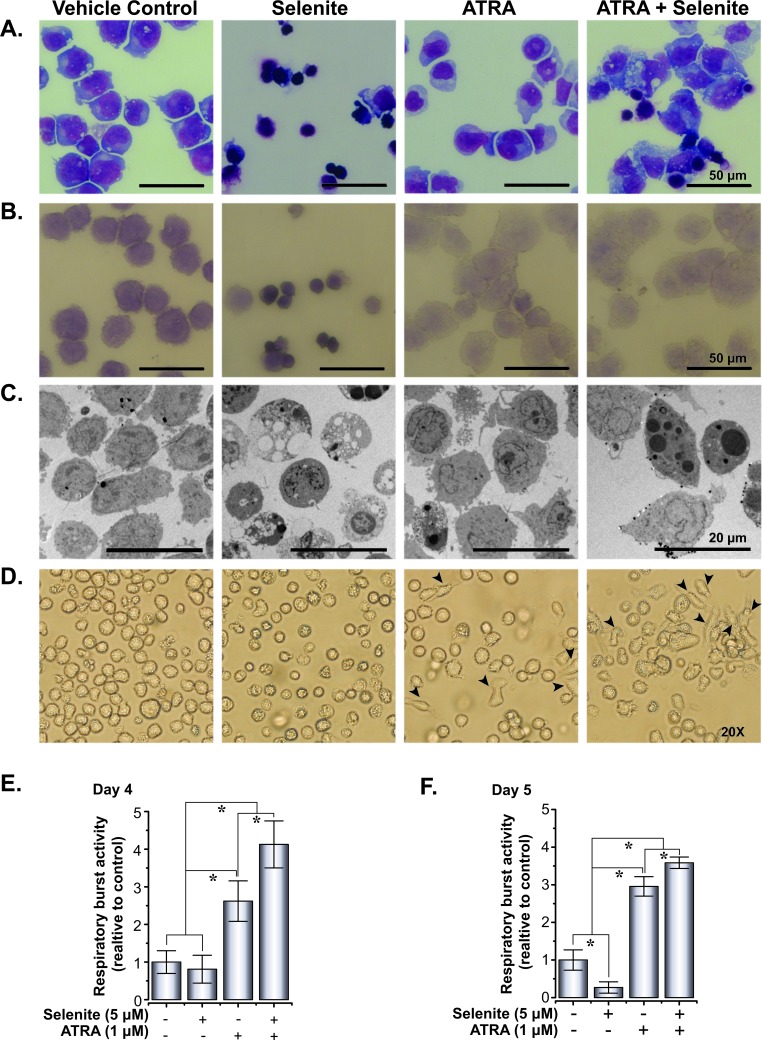
Selenite in combination with ATRA potentiates the maturation of NB4 cells **A.** Morphology of May-Grünwald-Giemsa stained cytospins of NB4 cells treated with selenite, ATRA or in combination. **B.** Myeloperoxidase staining of NB4 cells showing increased differentiated cell in ATRA and combined treatment following 5 days of treatment. **C.** Transmission electron micrograph of NB4 cells following 5 days of treatment. **D.** Phase contrast images of NB4 cells. Arrowheads indicate cells attached to culture vessels. Magnifications 20X. Images of the cells were acquired using an Olympus microscope (Model: IX73). **E.** and **F.** Functional assessment of differentiation in NB4 cells as evaluated by respiratory burst assay on day 4 and day 5.

Combined treatment with ATRA and ATO was previously shown to trigger differentiation in NB4 cells [21]. To address a similar mechanism of action by selenite alone or in combination with ATRA, we investigated differentiation in NB4 cells following treatment with these compounds in several ways. First, May-Grünwald-Giemsa staining of NB4 cells indicated augmented differentiation with varying morphology both in the ATRA and combined treatment (Figure 2A). In the combined treatment, majority of the cells were differentiated with a subset of dying cell population. Myeloperoxidase staining data indicated low staining intensities in these treatments, suggestive of advanced maturation status (Figure 2B). The differentiated cells exhibited characteristics bilobed or multilobular nuclei, dense neutrophilic granules and extensive vacuolization (Figure 2A and Figure 2C). We consistently observed an adherent sub-population of cells in ATRA and the combined treatment (Figure 2D). We furthermore measured the respiratory burst activity to evaluate the functional integrity of the differentiated cells. Respiratory burst activity was higher in the combined treatment than ATRA alone on both day 4 and 5, suggesting an advanced maturation status of cells receiving the combined treatment (Figure 2E and 2F). Significantly lower activities in the selenite treatment compared to control may reflect the poor cell viability after 120 h.

**Figure 3:**
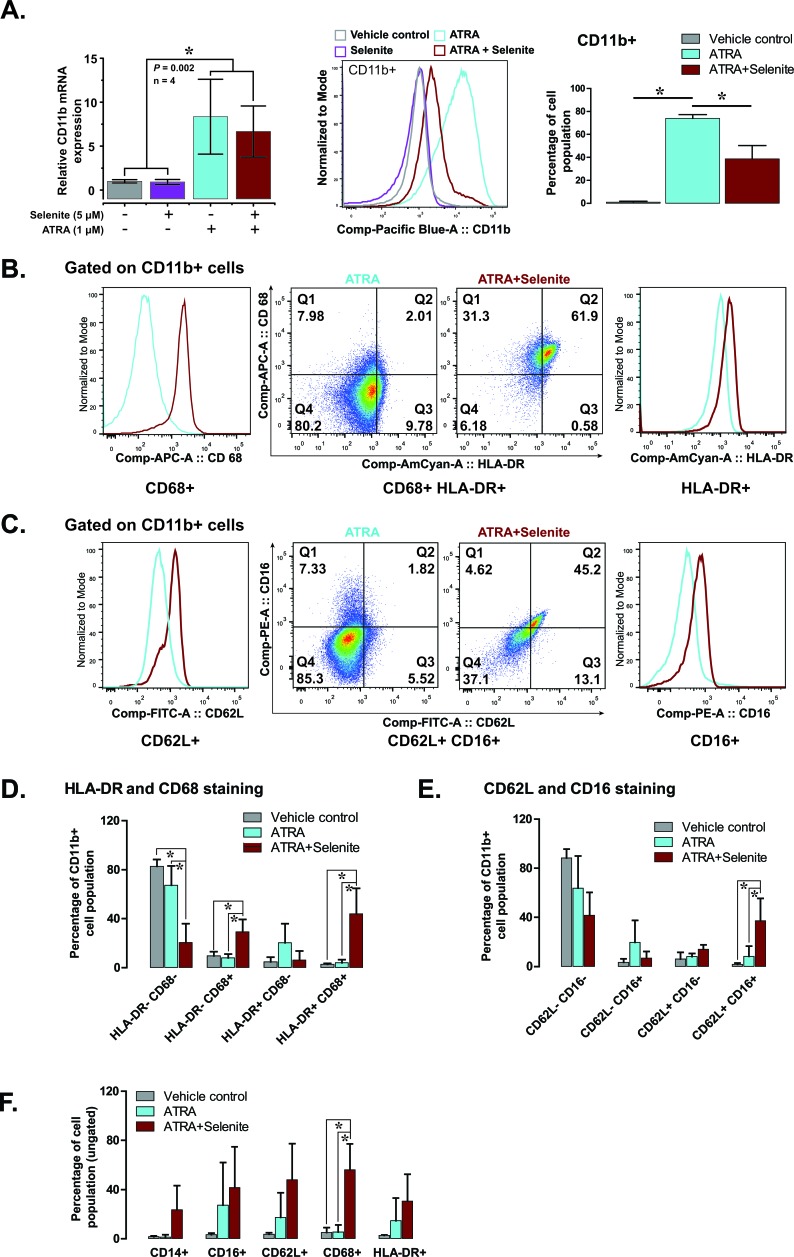
Selenite in combination with ATRA promotes the maturation of NB4 cells **A.** CD11b expression (mRNA in the left panel and protein in the middle and right panel) in NB4 cells following indicated treatment.**B.** & **C.** Representative flow cytometry analyses showing expression of CD68 and HLA-DR (markers of monocyte/macrophage lineage) and CD62L and CD16 (markers of neutrophil lineage) in CD11b positive NB4 cells. **D.** & **E.** Quantitative analyses of the expression of monocyte and macrophage lineage markers (HLA-DR+ and CD68+) and neutrophil lineage markers (CD62L+ and CD16+) in CD11b+ NB4 cells following treatment with selenite alone or in combination ATRA for 5 days (*n* = 3). **E.** Expression of individual markers in NB4 cells treated with selenite alone or in combination ATRA for 5 days (*n* = 3). **F.** Expression of CD14, CD16, CD62L, CD68 and HLA-DR in NB4 cells independent of their CD11b expression status.

Our observation of adherent cells was suggestive of possible differentiation of promyelocytes into monocytes or macrophages that display similar characteristics when cultured *in vitro*. To verify this, we carried out flow cytometry analyses of differentiation-associated cell surface markers to characterize their lineage. The expression of cell surface antigen CD11b was analyzed both at mRNA and protein level. ATRA and combined treatment resulted in a significant increase in CD11b mRNA level in comparison to the control and selenite treatments (left panel, Figure 3A). CD11b protein expression data (middle and right panel, Figure 3A) was congruent with the mRNA expression profile, albeit at a lower expression level in the combined treatment. Nevertheless, CD11b+ cells had higher expression of monocyte/macrophage lineage markers HLA-DR and CD68 in the combined treatment compared to ATRA alone (Figure 3B and 3D). A similar observation was made for neutrophil lineage markers CD62L and CD16 in the combined treatment (Figure 3C and 3E). The percentages of double positive cells were higher for both the cell lineages in the combined treatment (middle panel, Figure 3B and 3C). Together, these findings suggest advanced multi-lineage differentiation of NB4 cells upon combined treatment. The cluster of differentiation molecules expression supports the findings from the respiratory burst activity data, as outlined above. When the total cell populations (ungated) were analyzed for the expression of CD14, CD16, CD62L, CD68, and HLA-DR, corroborating results were found for all of these markers (Figure 3F). Although we could not demonstrate any differentiating effect of selenite as a single agent, the above findings provided the evidence for multi-lineage differentiation of NB4 cells following treatment with ATRA in combination with selenite.

### Selenite abrogates PML-RARα expression in NB4 cells

**Figure 4:**
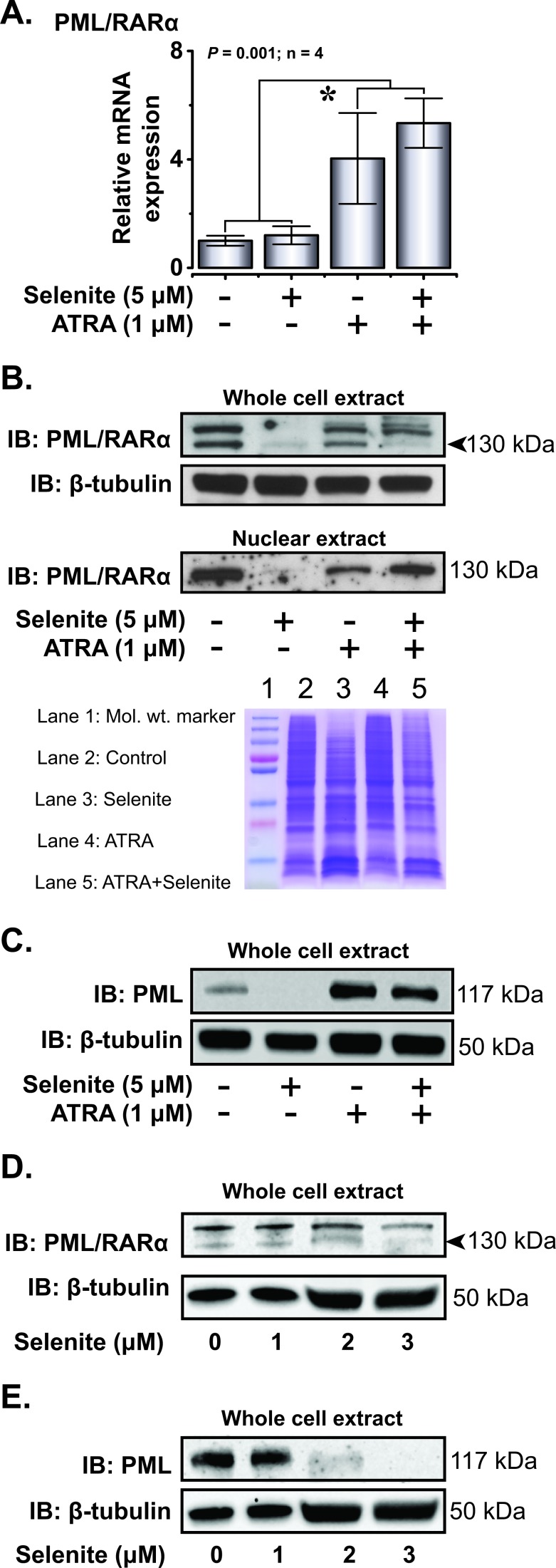
Selenite selectively degrades PML/RARα oncoprotein **A.** Relative PML/RARα mRNA expression in NB4 cells on day 4. **B.** Immunoblot analysis of PML/RARα in the whole cell extracts and nuclear fractions following treatment with the compounds (top panel). β-tubulin served as a loading control for the cytosolic fraction. Representative Coomassie blue stained gel as a loading control for PML/RARα in the nuclear extract (bottom panel). **C.** Immunoblot analysis of PML expression in NB4 cells. **D.** & **E.** Effects of increasing doses of selenite (1 - 3 μM) treatment on the degradation of PML/RARα and PML in NB4 cells.

Based on our preliminary hypothesis on thiol-modulating properties of selenite and subsequent suppression of PML/RARα, we proceeded to analyze the expression of PML/RARα both at mRNA and protein level in NB4 cells. Instead of suppression, we observed increased PML/RARα mRNA expression in cells receiving ATRA and the combined treatment, while selenite itself had no effect (Figure 4A). However, selenite treatment alone completely abrogated PML/RARα protein expression both in whole cell and nuclear extracts (Figure 4B), suggesting direct interaction of selenite with the PML/RARα protein. Consistent with the previous reports, the expression of PML/RARα was also reduced in the ATRA treatment. Notably, PML/RARα expression was somewhat restored in the combined treatment, suggesting potential interfering effects of ATRA on selenite-induced PML/RARα degradation. We also observed a similar effect on PML protein expression (Figure 4C). We further investigated the expression of native PML and PML/RARα proteins to test the hypothesis on abrogatory effect of selenite on zinc-thiolate coordination and subsequent loss of protein stability. We indeed observed dose-dependent (1.0 - 3.0 μM) abrogation of PML/RARα (Figure 4D) and PML (Figure 4E) expression by selenite.

### Regulation of differentiation-associated transcription factors by selenite and ATRA

**Figure 5:**
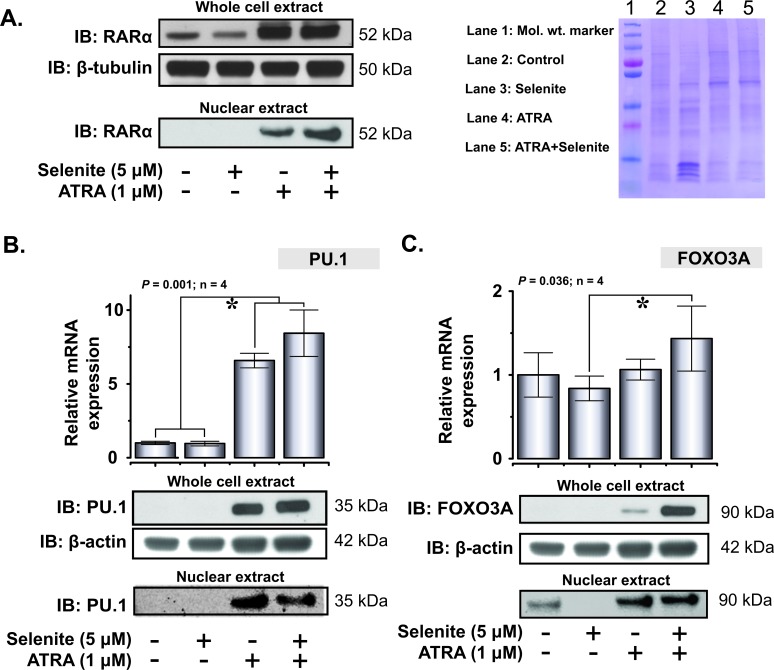
Restoration of expression of key differentiation-associated transcription factors in NB4 cells following ATRA or combined treatment with selenite **A.** Whole cell and nuclear expression of RARα following 5 days exposure to different treatments (top left panel). Representative coomassie blue stained gel as a loading control for the nuclear extract (top right panel). **B.** Relative mRNA expression of PU.1 (top panel). PU.1 expression in the whole cell and nuclear extracts in NB4 cells following single or combined exposure to ATRA and selenite (bottom panel). **C.** FOXO3A mRNA (top panel) and protein expression (bottom panel) in NB4 cells following treatment with ATRA, selenite or in combination. (*) indicates significant differences among the treatment groups.

Upon confirming the abrogation of PML/RARα protein expression by selenite, we subsequently investigated the expression of important transcription factors involved in the differentiation of the myeloid cell lineage. As reported previously, ATRA induced the expression of RARα in the whole cell extract [22] and co-exposure with selenite had no additional effects (Figure 5A). The expression pattern was similar in the nuclear extract, except for that the combined treatment resulted in higher expression of RARα compared to ATRA alone. We further investigated the expression of another important transcription factor PU.1, implicated in myeloid differentiation. Both ATRA and combined treatment significantly increased the mRNA level of PU.1 (Figure 5B). PU.1 protein expression both in the cytosolic and nuclear extracts corroborated well with the mRNA expression profile. Together, these results indicated that ATRA was necessary for augmentation of RARα and PU.1 expressions, while selenite alone had no priming effects. Furthermore, we found out that mRNA expression of FOXO3A was relatively stable with a robust increase in protein expression in the combined treatment (Figure 5C).

### Expression of thioredoxin and glutaredoxin family of proteins

**Figure 6:**
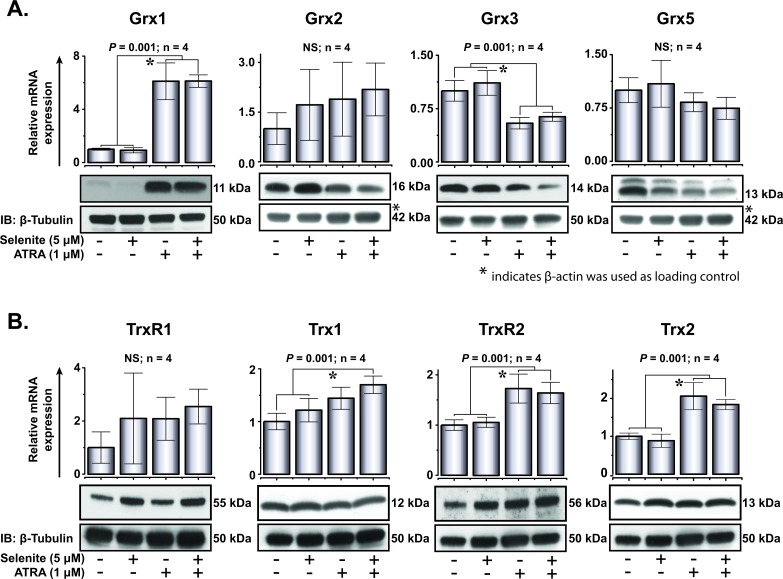
Expression of glutaredoxin and thioredoxin family of proteins following ATRA or combined treatment with selenite for 5 days **A.** Top, relative mRNA expression of Grx1, Grx2, Grx3 and Grx5. Bottom, immunoblot analyses of Grx1, Grx2, Grx3 and Grx5 protein expression (top panel) in NB4 cells. **B.** Top, relative mRNA expression of TrxR1, Trx1, TrxR2 and Trx2. Bottom, immunoblot analyses of TrxR1, Trx1, TrxR2 and Trx2 proteins expression (top panel) in these cells. * indicates significant differences among the treatment groups.

Proteins of the thioredoxin and glutaredoxin families play important roles in cytoprotection and multiple redox-dependent signaling pathways. One of the major drawbacks of ATO treatment in APL is the inhibition of thioredoxin reductase [23, 24], an enzyme that plays critical role in redox homeostasis both in health and disease. Earlier, it has been shown that the transcriptional activity of RARα is positively redox regulated by the selenoprotein thioredoxin reductase 3 [25]. With the above backgrounds in consideration, we explored the expression of thioredoxins and glutaredoxins in NB4 cells after treatment with ATRA and selenite. The mRNA expression of Grx1, Grx3 and Grx5 corroborated well with their protein expression (Figure 6A and 6B). Of these, the induction of Grx1 expression was remarkably higher in cells subjected to ATRA and the combined treatment. Among thioredoxins, TrxR2 and Trx2 were significantly upregulated both at mRNA and protein level in the differentiated cells compared to the untreated control cells (Figure 6B). There were minimal or no effects of selenite treatment on the expression of these important oxidoreductases independent of the differentiation status of the cells, apart from the selenoproteins TrxR1 and TrxR2.

## DISCUSSION

The PML/RARα oncoprotein functions as a transcriptional repressor [5] and thereby promotes malignant transformation and neoplastic progression [26] in APL with t(15;17) (q22;q21) chromosomal abnormalities [27, 28]. Selective degradation or blocking of its nuclear translocation abrogates its transcriptional activities accompanied by loss of its leukemogenic potential. To this end, the combined use of ATRA and ATO/chemotherapy presents a remarkable success in molecular targeted therapy [9, 29–31]. The critical cysteine residues in the zinc finger domain of the PML subunit of PML/RARα are potential therapeutic targets as disruption of this critical zinc/thiolate coordination site destabilizes its structural integrity, resulting in its degradation. The data presented in this study reveals that selenite, a simple inorganic form of selenium, abrogates the expression of PML/RARα in APL originated NB4 cells. In association with ATRA, it promotes multi-lineage differentiation in these cells that exhibit superior function in comparison to ATRA-induced differentiated cells.

In APL, inhibition of differentiation at the promyelocytic stage results in the exponential proliferation of blast cells. In accordance with other studies, we have also shown that ATRA at pharmacological concentration diminished cell proliferation, while cell survival remained unaffected. However, the combined treatment of ATRA with selenite resulted in diminished cell viability compared to ATRA alone, while higher percentages of the cell population underwent differentiation. Unlike a previous study [19], we failed to observe any potentiating effects of ATRA on the cytotoxicity of selenite in these myeloid originated cells. Rather ATRA treatment somewhat protected these cells against selenite cytotoxicity. This can be explained by diminished cell proliferation in the combined treatment. In our laboratory, we consistently observe reduced cytotoxicity of selenite at low cell densities due to corresponding low levels of extracellular thiols that determines selenium uptake from selenite and thereby its cytotoxicity [32]. Our study clearly demonstrates that cell density is one of the key determinants of selenite cytotoxicity *in vitro*.

We herein report superior phagocytic activity of the differentiated cells following the combined treatment. Increased frequency of differentiated cells (monocytes, macrophages and neutrophils lineage) provides the basis for the observed effects. From the perspective of any potential chemotherapeutic application, such findings may have important implications since the combined treatment not only induces cell death but also augments differentiation of promyelocytes into functionally active cells as determined by the respiratory burst activity.

While we have been pursuing our work, a parallel study has reported abrogation of PML/RARα protein expression following selenite treatment [33]. Consistent with this study, we also provide evidence for abrogation of PML/RARα expression by selenite alone in a different experimental set up. The partial repression of PML/RARα in the combined treatment with ATRA indicates the involvement of a process that may have limited the removal of zinc moiety from zinc/thiolate coordination of PML/RARα. It is plausible that inadequate selenium uptake, as could be inferred from the cytotoxicity data, may have been the limiting factor. On the other hand, the expression profile of important differentiation-associated transcription factors provides a reasonable explanation for the increased differentiation following the partial degradation of PML/RARα. A complex interplay among increased expression of key transcription factors RARα, PU.1 and FOXO3A presumably serve critical roles in the observed effects [7, 13]. Earlier, it has been reported that PML/RARα directly targets the PU.1 promoter, resulting in downregulation of PU.1 [4]. In spite of partial loss of PML/RARα, no differentiation blockade was observed in the combined treatment, thereby suggesting minimal interactions between PML/RARα and PU.1 at their transcriptional activities.

Recent evidence suggests that reactive oxygen species (ROS) act as signaling molecules in priming differentiation of common myeloid progenitor cells into mature cell types under normal physiological conditions in *Drosophila* [34]. Context wise, it is intriguing that AML cells exhibit higher expression of redox-regulatory proteins including thioredoxins and peroxiredoxins, coupled with an elevated basal ROS levels than their normal counterparts [35]. These observations suggest that the elevated ROS levels in myeloid malignancy may have different pathophysiological roles, possibly in ROS-mediated pro-survival signaling. No differentiation effect of selenite, a known ROS-inducing agent, is in line with the proposed paradigm. Our study also provides important information on the expression of key oxidoreductases both in undifferentiated and differentiated NB4 cells. Unlike ATO, we have found no inhibition of the expression of thioredoxin family of proteins following high doses of selenite treatment. Rather, a relatively increased expression of thioredoxins, both at mRNA and protein level, is evident following differentiation. Earlier, it has been shown that both murine and human monocytic leukemia cell lines overexpress glutaredoxins mRNA in response to experimental differentiation into macrophages [36]. In the present study, we report upregulation of Grx1 both at transcript and protein level in ATRA-induced differentiated cells without further increase by co-treatment with selenite. As Grx1 is a positive regulator of actin polymerization, migration, polarization and adhesion in neutrophils [37], its increased expression in differentiated cells indicates its potential functional involvement. However, the functional roles of reduced expression of Grx3 and Grx5 at protein level in these differentiated cells are not clear. Among the thioredoxin family of proteins, expression of TrxR2 and its substrate Trx2 were upregulated in the differentiated cells. This is an interesting observation given that ATRA is an inhibitor of the transcription factor NFE2L2 [38] that negatively regulates TrxR2 expression [39]. These findings are in contrast with ATO that exerts its inhibitory effects on redox regulatory proteins (e.g., TrxR1) through a direct interaction with the thiol groups of vicinal cysteine/selenocysteine residues located either in their catalytic sites or structural domains [24]. From the results of the present study, it is not clear whether these proteins are functionally involved in the myeloid differentiation or simply under transcriptional control by ATRA. However, our findings suggest that the expression levels of these proteins are altered following ATRA-induced myeloid differentiation, as reported elsewhere for both murine and human monocytic leukemia cells under experimental differentiation into macrophage-like cells [40].

**Figure 7:**
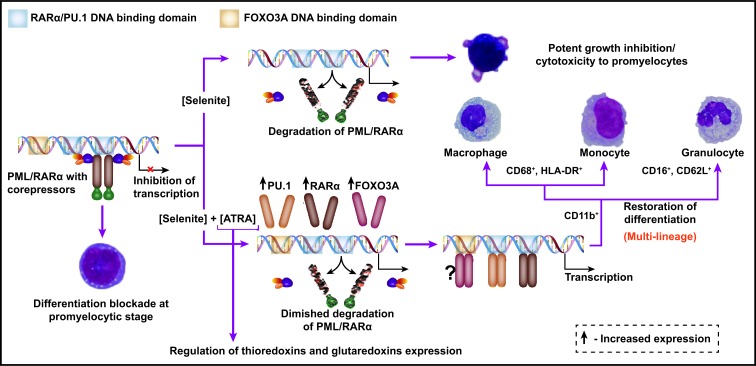
A schematic diagram depicting the combined use of selenite and ATRA as a novel therapeutic strategy for APL PML/RARα interacts with the shared DNA-binding domain of RARα and PU.1 transcription factors and thereby deregulates transcriptional control of terminal differentiation of promyelocytes. Selenite at pharmacological concentration abrogates PML/RARα protein expression and exerts growth inhibition and cytotoxicity to promyelocytes. In combination with ATRA, it partially degrades PML/RARα and induces the expression of RARα, PU.1 and FOXO3A transcription factors, all of which are important for myeloid differentiation. Cell surface antigen expression profile is suggestive of multi-lineage differentiation of these cells. Furthermore, ATRA-induced differentiation is accompanied by changes in the expressions of important oxidoreductases.

In summary, the present work reveals that pharmacological concentrations of selenite can selectively target PML/RARα oncoprotein for degradation. Combination of ATRA and selenite induces multi-lineage differentiation of APL cells, whilst a significant cell population undergoes apoptosis. This is depicted in a schematic diagram (Figure 7). The dose of selenite (5 μM), as used in the present study, is often considered too high to be used for pharmacological application in humans. To this end, findings from our previous clinical trial suggests that it is possible to reach much higher plasma selenium concentrations without any prolonged side effects and bone marrow toxicity in cancer patients following intravenous administration of selenite [17]. In fact, it was also shown that selenite at five-fold higher concentration than ATO at clinically relevant dose is much less toxic to normal bone marrow progenitor cells *ex vivo* [19]. These observations together with potent cytotoxicity of selenite to APL cells suggest prospective application of this compound in targeted therapy of APL that remains to be further investigated in clinical set up.

## MATERIALS AND METHODS

### Chemicals and antibodies

All the chemicals were purchased from Sigma-Aldrich, Germany unless mentioned otherwise. Antibodies for flow cytometry: BV421 Mouse Anti-Human CD11b/MAC-1, clone ICRF44; PerCP-Cy™5.5 Mouse Anti-Human CD14, clone M5E2; PE Mouse Anti-Human CD16, Clone B73.1; FITC Mouse Anti-Human CD62L, clone SK11 and BV421 Mouse IgG1, k Isotype Control, clone X40 were bought from BD Biosciences, Sweden. APC Mouse anti-human CD68, clone Y1/82A and AmCyan Mouse anti-human HLA-DR, clone L243 were bought from Biolegend, UK. Antibodies for western blot and their dilutions were as follows: RARα (Sc-551, C-20), 1:500, Santa Cruz Biotechnology; PML (A301-168A-1), 1:500, Bethyl Laboratories; PU.1 (GTX 45005), 1:1000, GeneTex; Grx1, 1:200, own production; Grx2 (GTX 112094), 1:1000, GeneTex; Grx3, 1:1000, own production; Grx5, (GTX 45005), 1:250, GeneTex; TrxR1 (GTX 110589), 1:1000, GeneTex; TrxR2 (ab58445), 1:750, Abcam; Trx1, 1:3000, own production; Trx2 1:3000, own production; Beta-actin (A4700), 1:1000, Sigma; Beta-tubulin (Ab6046), 1:3000, Abcam; FOXO3A (GTX 62705), 1:1000, GeneTex.

### Cell viability and proliferation

NB4 cells were obtained from the DSMZ (Braunschweig, Germany). Cells in early passages (3-10) were cultured in antibiotics-free RPMI 1640 media (Gibco, Paisley, UK) complemented with 10% heat-inactivated fetal bovine serum (FBS) (Gibco, Paisley, UK). For experimental purposes, cells were seeded at a density of 2.5×10^4^ cells ml^−1^ and treated with ATRA (dissolved in DMSO) and selenite (1.0 μM and up to 5.0 μM, respectively) either alone or in combination for 120 h. The final concentration of DMSO never exceeded 0.001%. To avoid nutrient deprivation-associated cell death, as reported earlier for NB4 cells [41], the media was replaced and treated alike after 72 h. At the end of the treatment, cell viability was measured using an automated cell counter (BioRad TC10, Stockholm, Sweden). Total cell count was used as a measure for cell proliferation. For apoptosis assay, FITC Annexin V Apoptosis Detection Kit I (BD Biosciences, Sweden) was used according to manufacturer protocol. For cell proliferation/cytotoxicity assay in 96-well plate, WST-1 reagent (Sigma-Aldrich, Germany) was used.

### Cytochemical staining

Following 120 h of treatment, cells were collected and resuspended in PBS containing 1% BSA. A total of 5.0×10^4^ cells were concentrated onto glass slides using Cytospin 3 (Shandon, Runcorn, UK) at 650 rpm, for 5 minutes. The air dried slides were stained with May-Grünwald and Giemsa. For myeloperoxidase (mPOX) staining, cells were left in the dark for at least 24 h and stained using a DAB substrate kit (Sigma-Aldrich, St. Louis, MO, USA) according to the manufacturer's protocol. Photomicrographs of the stained cells were acquired using a Nikon microscope (Eclipse E1000M) equipped with Nikon Digital Sight DS-Fi1 camera.

### Transmission electron microscopy

Following termination of treatments, cells were washed briefly with PBS and fixed in 2.5 % glutaraldehyde in 0.1 M phosphate buffer, pH 7.4 at room temperature for 30 min. After fixation, cells were rinsed in 0.1 M phosphate buffer and centrifuged. The pellets were then post-fixed in 2.0% osmium tetroxide in 0.1 M phosphate buffer, pH 7.4 at 4°C for 2 hour, dehydrated in ethanol followed by acetone and embedded in LX-112 (Ladd, Burlington, Vermont, USA). Ultrathin sections (approximately 50-60 nm) were cut by a Leica ultracut UCT/ Leica EM UC 6 (Leica, Wien, Austria). Sections were contrasted with uranyl acetate followed by lead citrate and examined in a Hitachi HT7700 (Tokyo, Japan) at 80 kV. Digital images were taken by using a Veleta camera (Olympus Soft Imaging Solutions, GmbH, Münster, Germany).

### Analyses of CD markers by Flow cytometry

At day 5 of treatment, cell surface expression of markers for monocytes, macrophages and neutrophils were analyzed using flow cytometry. Briefly, cells were washed twice with PBS containing 0.05% FBS (FACS buffer) and adjusted to equal numbers of cells for all the treatments. Five microliter of the antibodies was added to 100 μl cell suspensions and incubated for 20 min. Cells were washed once (1200 rpm for 5 min) with 500 μl of FACS buffer and resuspended in 200 μl FACS buffer. Subsequently, for each sample, at least 100000 events were acquired using a FACS Aria flow cytometer (Becton Dickinson, Sweden) using BD FACS Diva 6 software (Becton Dickinson, Sweden) and analyzed with FlowJo Software (Treestar Inc. OR, USA). Additional isotype control antibodies and fluorescence minus one control were used appropriately to aid the gating strategy while analyzing the data.

### Respiratory burst assay

As a measure of differentiation, respiratory burst assay was performed as described elsewhere [42] with these minor modifications. Cell suspensions were collected after 96 and 120 h and resuspended in the fresh RPMI 1640 medium supplemented with 10 % FBS. Total cell number were counted and diluted to a final concentration of 2.5×10^5^ live cells/ml (to compensate for cell death) and PMA was added to final concentration of 200 ng/ml. Subsequently, triplicates of 100 μl cell suspension aliquots were transferred to 96-well microplates and 10 μl of WST-1 was added to each well immediately. The plate was incubated for 1 h and the absorbance was measured at 412 nm.

### Quantitative real-time PCR

After 96 h of treatment, total RNA was extracted by using RNeasy Plus Mini kit (Qiagen, Stockholm, Sweden) with on column DNase digestion, following the manufacturer protocol. RNA content in each sample was quantified by using a Nanodrop^®^ Spectrophotometer ND-1000, followed by cDNA synthesis using Omniscript Reverse Transcription Kit (Qiagen, Sweden) containing 2 μg RNA from each sample and 0.1 μg/μL Oligo(dT)_12-18_ as primer. Real-time PCR was performed in a CFX96^TM^ Real-Time System (Bio-Rad, Stockholm, Sweden) using SyberGreen Supermix (Bio-Rad, Stockholm, Sweden), 20 ng of cDNA per reaction in a final volume of 10 μl and primer concentration ranging from 300-900 nM (primers sequence available upon request). The data were analyzed using the 2^−ΔΔCT^ method. The expression level of HPRT was used to normalize the relative expression levels of the genes of interest.

### Immunoblotting

At day 5 of treatment, NB4 cells were collected and washed twice with PBS. Whole cell lysates were prepared using RIPA buffer (Sigma-Aldrich, St. Louis, MO, USA) containing PMSF (1 mM, final concentration) and protease inhibitor cocktail (Sigma-Aldrich, St. Louis, MO, USA). Nuclear extract was prepared using a hypotonic buffer solution [43]. Lysates protein content was determined by Bio-Rad protein assay kit. A total of 25-50 μg of protein was loaded per well and separated on SDS-PAGE gel (Bio-Rad, Stockholm, Sweden) and transferred to a 0.20/0.45 μm pore-sized PVDF membrane (Bio-Rad, Stockholm, Sweden). After transfer, the membrane was incubated with primary antibodies overnight at 4°C followed by horseradish peroxidase-conjugated secondary antibodies for 1 h at room temperature. Enhanced chemiluminescence (ECL, Perkin Elmer) was used for development of the blot. The antibodies used and their dilutions are available in the supplemental data.

### Statistical analyses

All data have been presented as mean ± SD (N, sample size), unless stated otherwise. Assumptions of parametric statistical tests (i.e., equality of normality and variance) were tested prior to data analysis. Equivalent non-parametric tests were employed when data did not follow the assumptions of parametric tests. Pairwise comparison was performed using student's *t*-test. For multiple comparisons analysis, one-way ANOVA followed by Tukey's multiple comparison tests (Dunn's multiple comparison for non-parametric test) was employed. Mean value was considered significantly different at *P* < 0.05. All the statistical tests were performed using SigmaPlot, Version 11.

## Abbreviations

APL: Acute Promyelocytic Leukemia
ATO: Arsenic trioxide
ATRA: All-*trans* retinoic acid
FOXO3A: Forkhead box protein O3A
GPX: Glutathione peroxidase
Grx: Glutaredoxin
HDAC: Histone deacetylase
PML: Promyelocytic leukemia protein
PU.1: Transcription factor PU.1
RARα: Retinoic acid receptor alpha
Trx: Thioredoxin
TrxR: Thioredoxin reductase
